# Telemedicine Intervention to Improve Long-Term Risk Factor Control and Body Composition in Persons with High Cardiovascular Risk: Results from a Randomized Trial: Telehealth strategies may offer an advantage over standard institutional based interventions for improvement of cardiovascular risk in high-risk patients long-term

**DOI:** 10.5334/gh.825

**Published:** 2021-03-25

**Authors:** Nana Pogosova, Yulia Yufereva, Olga Sokolova, Anara Yusubova, Alexander Suvorov, Hugo Saner

**Affiliations:** 1 Federal State Institution “National Medical Research Center of Cardiology” of the Ministry of Healthcare of the Russian Federation, Moscow, RU; 2 Children’s Diagnostics and Treatment Center after N.A. Semashko, Moscow, RU; 3 City Hospital #4, Moscow, RU; 4 Institute for Social and Preventive Medicine, University of Bern, Bern, CH

**Keywords:** risk factors, high cardiovascular risk, physical inactivity, unhealthy nutrition, smoking, obesity, anxiety, depression, preventive counseling, telemedicine technology

## Abstract

**Background:**

Telehealth strategies are increasingly used to support people at high cardiovascular risk long-term, but is it unclear if these interventions are effective at improving cardiovascular risk.

**Objective:**

To evaluate the effects of a telemedicine technology-based program on risk factor control and body composition in patients at high cardiovascular risk.

**Methods:**

This is a population based randomized controlled trial. 100 patients at high and very high cardiovascular risk were randomly assigned to a telemedicine technology-based program consisting of: Comprehensive counseling on risk factors delivered by a physician; biweekly remote support via phone delivered by a trained nurse during the first three months after enrollment; and a control group receiving routine care with individual single-session counseling on patients’ current risk factors without further support. The follow-up period was 1 year.

**Results:**

Mean age of participants was 59.9 ± 4.5 years, 80% were women. Weight (–0.582; p < 0.001), waist circumference (–0.429; p = 0.01), body mass index (–0.216; p < 0.001) diastolic blood pressure (–0.881; p = 0.04), total cholesterol (–0.149; p = 0.01) and LDL cholesterol (–0.123; p = 0.003) were lower in the intervention group compared to the control group after 12-month. Body fat mass was also lower (–0.352; p = 0.01) and lean mass was higher (0.92; p = 0.03) in the intervention group. Anxiety scores (–2.5; p < 0.002) and depression scores (–2.6; p < 0.001) were also lower in the intervention group.

**Conclusions:**

Among older people at high cardiovascular risk, the addition of telehealth strategies using remote support by phone calls over a period of 3 month resulted in small but significant improvements of cardiovascular risk factors, body composition, anxiety, and depression which are maintained long-term. Such telehealth strategies may offer an advantage over standard institution-based interventions.

## Introduction

Cardiovascular disease (CVD) is the leading cause of morbidity and mortality in most countries around the world including Russia [1]. Patients with a high CVD risk often present with excess weight, hypertension, and dyslipidemia. The negative impact of most CVD risk factors can be reduced by adopting a healthy lifestyle with adequate physical activity, a healthy diet, and without smoking [2,3]. Institution-based behavioral counseling and lifestyle intervention is the most commonly used approach to tackle this situation [4,5]. However, availability of such comprehensive programs is limited, and there are many barriers for participation in such programs including access, social and cultural attitudes, poverty, and environmental factors. There have been effective community-based programs aimed at reducing cardiovascular risk factors in low- and middle-income countries but these have been generally limited to the urban poor [6]. Furthermore, the growing prevalence of persons with obesity and cardiometabolic risk factors means that new and more efficient approaches to healthcare delivery are needed to support people in managing their own care, preferably with less reliance on institutional consultations by expensively trained health care professionals.

Facilitating patients’ access to care by using telehealth technologies is an important strategy to address this challenge. Many countries are exploring greater use of telehealth technologies as a way to expand the provision of care and to increase access to care for a large number of people at relatively low cost. The number of publications on the effectiveness of specific telehealth interventions including internet, remote monitoring, and telephone support is rapidly increasing [7,8,9,10,11,12,27,28]. However, recent reviews have highlighted poor quality of evidence due to inconsistency of methods and results [13,14]. Whereas short-term effects are usually found to be positive, there is a lack of results during longer-term follow-up.

The aim of this trial is to evaluate the effects of a telemedicine technology-based program on risk factor control, body composition, anxiety, and depression in patients at high and very high cardiovascular risk over a period of one year.

## Methods

### Study Design

This is a randomized controlled clinical trial which has been conducted in high cardiovascular risk individuals. We examined the effect of a telemedicine technology-based program on patients’ cardiovascular risk profile and body composition. Subjects were randomly assigned in a parallel design (1:1) to either an intervention or a control group. Participants assigned to the intervention group received comprehensive counseling on risk factors–with a main focus on nutrition–delivered by a physician followed by biweekly remote support by phone provided by a trained nurse during the first three months after enrollment (six sessions). The participants in the control group received a routine individual single-session counseling on their current risk factors in health centers. The structure and content of this counselling session were at the discretion of the health center physician. Due to the high cardiovascular risk, all participants were also eligible for the state-run yearly health check-up program encompassing comprehensive assessment and management of their current risk factors profile which also includes counselling. Follow-up evaluations were performed after 6 and 12 months.

### Participants

All patients visiting two community-based health centers in Moscow who were considered eligible during the routine health-center check-up were invited to participate. Those who agreed signed the informed consent form and proceeded with the randomization. Participants have been included in the study according to the following criteria: Men and women aged 40 to 65 years with high or very high cardiovascular risk defined as 5–9% and ≥ 10% according to the Systematic Coronary Risk Evaluation (SCORE) scale for countries with high cardiovascular mortality [15]; and two criteria for metabolic syndrome according to the Russian national guidelines for CVD prevention [4], where metabolic syndrome is defined as the presence of one major criteria (abdominal obesity with waist circumference ≥ 94 cm in men or ≥ 80 cm in women) and two minor criteria defined as blood pressure ≥ 140/90 mmHg; serum triglycerides > 1.7 mmol/L; HDL-cholesterol < 1.0 mmol/L in males or < 1.2 mmol/L in females; LDL-cholesterol > 3 mmol/L; fasting glucose > 6,1 mmol/L; and 2-hour glucose after oral glucose tolerance test ≥ 7.8 and ≤ 11.1 mmol/L. The decision to use the Russian metabolic syndrome criteria for obesity in addition to SCORE was based on alarmingly growing prevalence of obesity and associated health risks in Russia. Exclusion criteria were based on the participant’s medical records and included previous diagnosis of any clinical atherosclerotic disease (coronary artery disease, cerebrovascular disease, or peripheral artery disease), diabetes mellitus (fasting blood glucose equal to or higher than 7 mmol/L or any non-fasting blood glucose ≥ 11.1 mmol/L or the 2-hour blood glucose of ≥ 11.1 mmol/L for the oral glucose tolerance test), life-threatening arrhythmias, heart failure, renal or hepatic failure, active cancer, uncontrolled asthma, mental diseases, alcohol or substance abuse. Other exclusion criteria included inability to get counseling or to fill in questionnaires due to insufficient knowledge of the Russian language or illiteracy.

The randomization was stratified by sex (male vs. female) and age (<55 vs. ≥55 years old). Random numbers generated by a computer were used and the enrolled participants were randomized in a 1:1 ratio into two parallel groups.

All participants provided written informed consent. The study protocol has been approved by the Institutional Ethics Committee of the National Center for Preventive Medicine, Protocol number #02-13/15.

### Intervention

**Figure 1:**
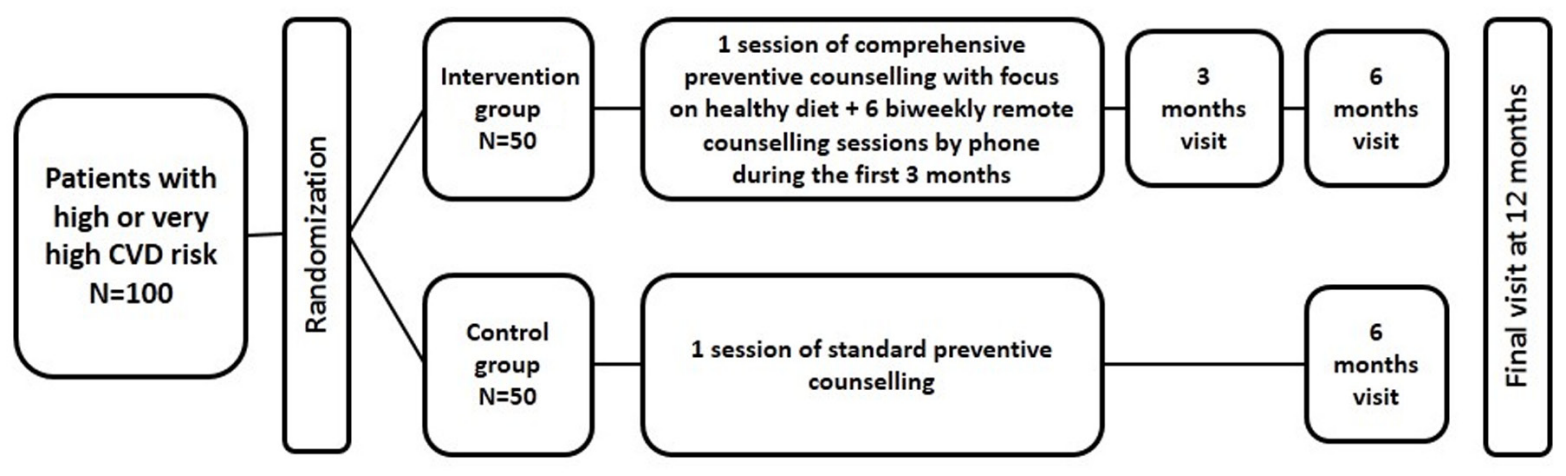
Flow chart of study procedures.

A schematic representation of the intervention and study-specific procedures is shown in Figure 1.

The intervention was a telemedicine technology-based program consisting of one face-to-face comprehensive counseling on cardiovascular risk factors with focus on nutrition in an out-patient health center, followed by 3-months of remote counselling sessions provided via phone in a biweekly regimen. The face-to-face counseling was provided by a physician in a structured manner during 80–90 minutes which included an informational section, a discussion, and a check of the acquired knowledge. The information provided to patients included the concept of cardiovascular risk with focus on the SCORE scale and its components [15], detailed discussion of traditional risk factors, discussion on what high/very high CVD risk means (not yet a disease, but no longer healthy), evaluation of reasons for unhealthy behavior, motivation of the patients towards risk reduction, setting individual specific realistic goals, education on self-monitoring of risk factors (including training in measuring of waist circumference, calculating body mass index, keeping a food diary, estimating daily energy expenditure and daily caloric intake), counselling on food choices and physical activity, and motivation of the patients to adhere to the prescribed medications. The information on a healthy diet was based on the contemporary national guidelines on CVD prevention [4] which were consistent with the 2012 version of the ESC guideline [16]. During the following three months, biweekly remote support was provided by phone calls from a trained study nurse. The telephone contact comprised of a semi-structured conversation about the participant’s current dietary habits such as: number of meals per day; the length of time between the last meal and going to bed; vegetable, fruit and fish intake; salt and high-sodium food restriction; added sugar intake; habitual physical activity; and medication adherence. After discussing patient-specific goals, results, and difficulties, the nurse provided advice and set out new goals for further improvement. Follow-up measurements were performed at 6 and 12 months after enrollment for both the intervention and the control groups. The intervention group had an extra follow-up visit at 3 months in order to assess short-term effects of the intervention and to reduce disappearance for the longer term.

### Measurements

At the first visit, all participants filled in a questionnaire giving information on gender, age, educational level, as well as professional and family status. Height and weight were taken in light indoor clothes without shoes using SECA scales 701 and measuring stick model 220. BMI was calculated by weight divided by squared height. Waist circumference was measured using a metal tape applied horizontally at the point midway in the mid-axillary line between the lowest rim of the rib cage and the tip of the hip bone (superior iliac crest) with the patient in standing position. Fat mass was estimated using whole-body electrical impedance analysis at 5 and 50 kHz between the hand and foot with the Impedance Analyzer ABC-01 “MEDASS”, software version ABC01-036 “MEDASS” (MEDASS, Russia). The body impedance analysis was carried out in the supine patient’s position, no less than 1 hour after the last meal. Blood pressure (BP) was measured in sitting position after 5 minutes rest using an Omron M6 automatic digital sphygmomanometer. Two BP measurements were taken at the right upper arm with a 5-minute interval, and the mean value was used. Blood cholesterol and glucose were measured under fasting conditions using a CardioCheck PA® point-of-care device (Polymer Technology Systems, USA) at all study visits. During the first and the final visits, venous (fasting) blood was drawn in addition to the other measurements to control for serum total and high-density lipoprotein (HDL) cholesterol and triglycerides. Low-density cholesterol (LDL) was calculated according to Friedewald’s formula. Physical activity was assessed using the IPAQ questionnaire [17]. Anxiety and depressive symptoms were assessed using the Hospital Anxiety and Depression scale [18]. The primary outcome parameters were changes of traditional CVD risk factors and body composition.

### Statistical analysis

The distributions of baseline characteristics of the participants were examined for the intervention and the control group. Means and SDs were calculated for continuous variables, and proportions were calculated for categorical variables. To examine baseline differences between the intervention and control group, Student’s t-test was used for continuous variables and the Chi2 test was used for categorical variables.

Linear mixed-effects models were used to examine changes in dependent variables over time of follow-up. Groups and observation time were considered as fixed effects; changes between groups and group-time interaction models were assessed using ANOVA. Statistical analysis was performed in R version 3.6 [R Core Team (2020), R: A language and environment for statistical computing. R Foundation for Statistical Computing, Vienna, Austria. URL https://www.R-project.org/].

## Results

**Figure 2:**
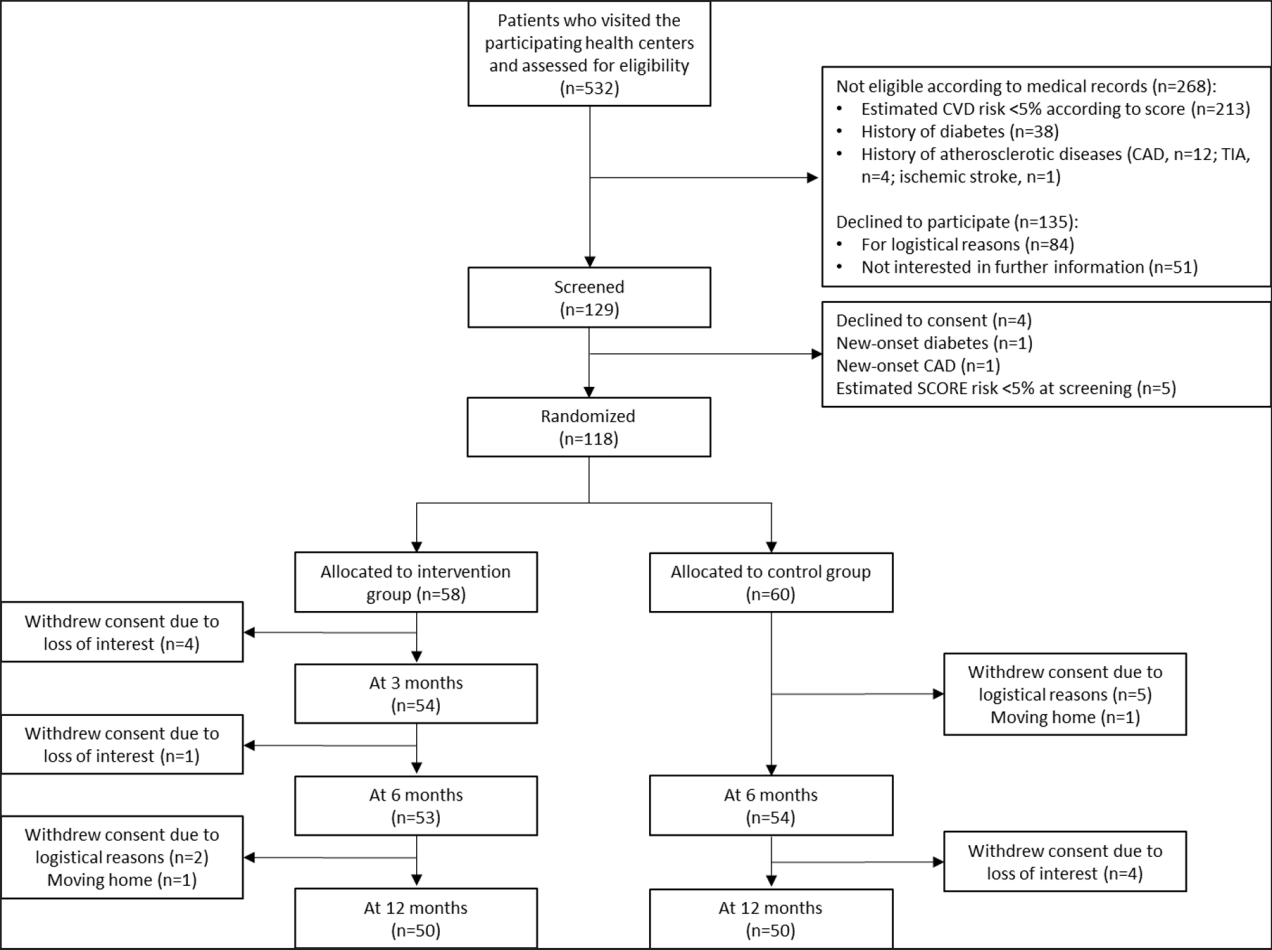
Flow chart of study participants.

The flow of study participants during the study procedures is shown in Figure 2. In total, 532 patients were assessed for eligibility, of which 268 were not eligible because of the presence of exclusion criteria according to their medical records and 135 (25.4%) declined to participate. Of 129 eligible patients, 11 were not included due to different reasons, most due to their CVD risk at screening being lower than previously recorded or to patients’ refusal. 118 participants were randomized: 58 patients were allocated to intervention group and 60 patients to the control group. During the study period, 18 patients were lost to follow-up due to loss of interest and/or change of residence. Follow-up was stopped at 341 ± 16 days when the final number had reached 50 participants for each group. The current analysis is based on those who completed the program (completers). There were no deaths, hospital admissions, or other serious events during the follow-up period. Mean age of completers is 59.9 ± 4.5 year, 80% were woman.

The baseline characteristics of patients are presented in Table 1. Due to the randomization being stratified only by age and gender, small differences were found between study groups at baseline with regard to total cholesterol, triglycerides, and fasting blood glucose. The high proportion of women and of participants with higher education is very typical for the health centers statistics showing higher interest in health education in these categories.

**Table 1:** Baseline characteristics of study groups.

	Intervention group (n = 50)	Control group (n = 50)	*p-value*

*Demographics*			
Age, years	59.7 (4.9)	60.0 (4.0)	0.67
Women, n (%)	41 (82.0)	39 (78.0)	0.62
Educational level			0.64
Secondary education, n (%)	3 (6.0)	1 (2.0)	
College, n (%)	17 (34.0)	14 (28.0)	
Incomplete higher education, n (%)	1 (2.0)	1 (2.0)	
Higher education, n (%)	29 (58.0)	34 (68.0)	
Body weight, kg	82.0 (14.0)	81.8 (11.0)	0.96
Body mass index, kg/m^2^	30.6 (4.1)	30.2 (4.0)	0.62
Waist circumference, cm	96.3 (10.2)	95.9 (8.7)	0.82
Fat mass, kg	29.6 (7.8)	29.3 (7.5)	0.85
Lean mass, kg	52.4 (10.3)	52.4 (9.2)	0.99
Systolic BP, mmHg	148.21 (20.0)	147.7 (19.0)	0.93
Diastolic BP, mmHg	85.6 (9.5)	84.9 (8.8)	0.71
Total cholesterol, mmol/l	6.4 (1.2)	5.9 (0.9)	**0.04**
HDL cholesterol, mmol/l	1.5 (0.5)	1.5 (0.3)	0.59
LDL cholesterol, mmol/l	4.2 (1.0)	4.0 (0.8)	0.33
Triglycerides, mmol/l	1.7 (0.9)	1.4 (0.5)	**0.03**
Fasting blood glucose, mmol/l	5.5 (0.6)	5.2 (0.7)	**0.04**

*BP* blood pressure; *HDL* high density lipoprotein; *LDL* low density lipoprotein. Numbers are mean (SD), unless otherwise stated.

Over 12 months, there was a small but significant improvement in a number of key CVD risk factors in the intervention group as compared to the control group.

Using linear mixed-effect models, improvements were seen for most of the study parameters including body weight, BMI, waist circumference, and diastolic blood pressure over time. Also observed were a significant increase in metabolic equivalent of walking minutes and total metabolic equivalent of activity minutes.

Table 2 shows between and within study group comparisons of cardiometabolic risk factors during follow-up. Diastolic blood pressure (–0.881; p = 0.04), total cholesterol (–0.149; p = 0.01), and LDL cholesterol (–0.123; p = 0.003) were significantly lower in intervention group compared to control group. Within the whole cohort, systolic (–4.726; p < 0.001) and diastolic blood pressure (–0.709; p = 0.02) decreased over the 12-month period, whereas a minimal increase of fasting blood glucose values was observed (+0.055; p = 0.05).

**Table 2:** Between and within study groups comparison over time for body composition.

Measurement		Timeline				Group		Time		Group × Time	*p*-value

		Baseline	3 months	6 months	12 months	Est.	*p*-value	Est.	*p*-value	Est.	

*Weight*	Intervention	82.0 ± 14.0	79.6 ± 14.0	81.4 ± 10.9	81.5 ± 11.1	0.286	0.90	–0.126	0.216	**–0.582**	**<0.001**
	Control	81.8 ± 11.0	NA	79.8 ± 14.1	79.5 ± 13.6						
*Waist circum*.	Intervention	96.3 ± 10.2	94.2 ± 10.0	94.3 ± 10.5	94.3 ± 10.2	0.406	0.82	–0.147	0.24	**–0.429**	**0.01**
	Control	95.9 ± 8.7	NA	95.6 ± 8.5	95.4 ± 8.7						
*BMI*	Intervention	30.6 ± 4.1	29.7 ± 4.0	29.8 ± 4.3	29.7 ± 4.1	0.268	0.73	–0.045	0.24	**–0.216**	**<0.001**
	Control	30.2 ± 3.4	NA	30.1 ± 3.4	30.1 ± 4.1						
*Fat mass*	Intervention	29.6 ± 7.8	27.4 ± 7.5	27.8 ± 8.1	28.0 ± 7.7	–0.471	0.74	–0.090	0.37	**–0.352**	**0.01**
	Control	29.3 ± 7.5	NA	29.1 ± 7.4	29.0 ± 7.6						
*Lean mass*	Intervention	52.4 ± 10.3	52.2 ± 10.6	52.0 ± 10.4	51.5 ± 9.8	0.924	0.41	–0.029	0.73	**–0.258**	**0.03**
	Control	52.4 ± 9.2	NA	52.2 ± 9.1	52.3 ± 9.2						

*BMI* body mass index. *NA* no measurements taken at time point. Values are expressed as mean (SD). Models are adjusted for sex and age.

Table 3 shows the results for body composition measurements in study groups during follow-up. The intervention group showed a significantly lower body weight (–0.582; p < 0.001), waist circumference (–0.429; p = 0.01), BMI (–0.216; p < 0.001), fat mass (–0.352; p = 0.01), and lean mass (–0.258; p = 0.03) compared to the control group over the 12-month follow-up period. Values for physical activity and related metabolic equivalents showed small but insignificant improvements in the intervention group compared to the control group. Values for anxiety scores (–2.5; p = 0.002) and depression scores (–2.6; p = 0.009) were slightly lower in the in the intervention group compared to the control group.

**Table 3:** Between and within study groups comparison over time for cardiometabolic risk factors.

Measurement		Timeline				Group		Time		Group × Time	

		Baseline	3 months	6 months	12 months	Est.	*p*-value	Est.	*p*-value	Est.	*p*-value

*SBP*	Intervention	148.1 ± 19.0	130.3 ± 9.3	131.0 ± 12.4	130.3 ± 13.2	–3.247	0.36	**–4.726**	**<0.001**	–0.530	0.59
	Control	147.7 ± 20.0	NA	135.3 ± 13.2	134.3 ± 14.2						
*DBP*	Intervention	85.6 ± 8.8	79.7 ± 6.5	80.6 ± 7.3	79.9 ± 8.2	–0.005	0.99	**–0.709**	**0.02**	**–0.887**	**0.04**
	Control	84.9 ± 9.5	NA	83.6 ± 7.8	82.7 ± 7.6						
*Total cholesterol*	Intervention	6.4 ± 1.2	6.5 ± 1.3	6.5 ± 1.5	5.9 ± 1.1	**0.660**	**0.01**	–0.013	0.75	**–0.149**	**0.01**
	Control	5.9 ± 0.9	NA	6.2 ± 1.1	5.8 ± 1.0						
*HDL cholesterol*	Intervention	1.5 ± 0.5	NA	NA	1.4 ± 0.5	0.053	0.56	–0.008	0.46	–0.015	0.32
	Control	1.5 ± 0.3	NA	NA	1.5 ± 0.3						
*LDL cholesterol*	Intervention	4.2 ± 1.0	NA	NA	3.7 ± 0.9	0.308	0.15	–0.029	0.32	**–0.123**	**0.003**
	Control	4.0 ± 0.8	NA	NA	3.9 ± 1.0						
*Triglycerides*	Intervention	1.7 ± 0.9	NA	NA	1.7 ± 0.7	**0.367**	**0.02**	–0.011	0.64	0.010	0.77
	Control	1.4 ± 0.5	NA	NA	1.3 ± 0.4						
*Fasting BG*	Intervention	5.5 ± 0.6	5.5 ± 0.7	5.5 ± 0.6	5.6 ± 0.5	**0.286**	**0.04**	**0.055**	**0.05**	–0.023	0.54
	Control	5.2 ± 0.7	NA	5.3 ± 0.5	5.4 ± 0.6						

*BG* blood glucose; *DBP* diastolic blood pressure; *HDL* high density lipoprotein; *LDL* low density lipoprotein; *SBP* systolic blood pressure. *NA* no measurements taken at time point. Values are expressed as mean (SD). Models are adjusted for sex and age.

## Discussion

This trial demonstrated that a telemedicine technology-based intervention program for patients at high and very high cardiovascular risk, starting with an initial comprehensive counselling intervention followed by telephone counselling over a three-month period, can lead to small but statistically significant improvements in a number traditional CVD risk factors as well as in anthropometric and body composition measures. Positive changes were sustained at 12 months of follow-up.

Whereas there is a wealth of data regarding the benefits of cardiovascular risk factor counselling in the general population, results for high and very high-risk patients are rare and have usually been evaluated only in the short term. Behavioral counselling provides small but significant benefits in healthy people, including reductions in blood pressure and low-density lipoprotein cholesterol levels as well as improvements in measures of adiposity. Persons who are interested or ready for behavioral changes may be the most likely to benefit. Nevertheless, the ESC guidelines for CVD prevention [5] state that there is limited evidence to determine which interventions are the most effective in specific groups. A review of community-based interventions for prevention of CVD in low- and middle-income countries involving population-based and high-risk interventions showed limited effectiveness of interventions such as: Education of patients, training of health care providers, and implementing treatment guidelines [6]. As the obesity prevalence is on the rise in Russia, like in many other developed countries [8], more efficient approaches to healthcare delivery are needed to support people to manage their own care. Therefore, obesity and related metabolic risk factors were targeted when designing the counselling program. This trial was performed in a pragmatic way to find the most appropriate approach for the relatively unselected population of high-risk patients visiting community-based health centers. Additionally, the intervention utilized in this study contains a telemedicine component, following the ESC recommendation ‘to support research into the development, evaluation, and implementation of e-health technologies, with an emphasis on establishing the clinical and cost-effectiveness of such innovation, and including the patient perspective’ [19].

The spectrum of risk factors that showed improvement over 12 months with the intervention utilized in this study is consistent with previous research. Previous meta-analyses have shown a reduction of adiposity parameters [20,21], total cholesterol and LDL cholesterol [22], and blood pressure control [23]. Nevertheless, the magnitude of these positive effects was distinctly less in this study compared to results from the meta-analyses cited above.

However, the overall effects of this telehealth intervention show good internal consistency: The reduction of the adiposity parameters and blood lipids were in line with a number of more healthy eating patterns in the intervention group, which have been previously reported [24,25]. More specifically, the intervention group reduced their intake of sweets and chocolate, sausages or smoked meats and high-fat cheese, increased the time between the last meal and bedtime, and reduced the average number of meals per day.

The fact that not only anthropometric adiposity measures but also body fat percentage by bioelectrical impedance analysis was reduced, seems of particular interest in view of the results of the recently published PREVEND study [26]. This cohort study demonstrated that body fat percentage by bioelectrical impedance analysis was not only independently associated with CVD events in men and women, but it was actually superior to BMI and waist circumference with respect to discrimination ability and additive predictive value. These are important messages pertinent to the prognostic value of body fat measured by impedance analysis. The reduction of body fat seen in this study in the comprehensive counselling and remote support group may also indicate the potential of this intervention to improve long-term outcomes.

The observed changes of blood pressure can most likely be attributed to the observed adiposity reduction and salt restriction since neither the number of antihypertensive drugs nor the self-reported drug adherence differed significantly between groups at 12 months of follow-up.

The strength of the study is the high completion rate in both intervention and control group. Although 18 participants were lost to follow-up, the number of missing data is relatively low. The combined evaluation of metabolic factors with measurements of body composition is another strength of the study procedure which contributes to the positive interpretation of the overall results. Furthermore, the approach to a comprehensive risk factor intervention is relatively simple and inexpensive and can easily be integrated in usual clinical practice–in particular in countries with limited internet access or limited health care resources. Nevertheless, there may be other applications of telehealth interventions besides telephone counselling and remote support such as web or mobile technologies-based programs. The integration of such interventions may be more successful. Further studies are needed to better understand the full potential of telehealth interventions.

There are several limitations to this study. One limitation is the sample size. The study sample represents a pragmatic sample of older adults, and the results cannot be generalized to other age groups. The context of this intervention is that participants came from a community-based health-care center, which may represent a selection bias. Another limitation is the fact that 60% of participants had higher education which limits the generalizability of the results in regard to effects which may be achieved in a less educated population. Another limitation is that one cannot exclude that the extra follow-up visits at three months diluted the effects of the telehealth consultations. A particularity of this telehealth intervention were telephone calls by specially trained nurses over a period of three months, which has to be considered when interpreting the results. Other telehealth technologies or combinations of such technologies may lead to different results. Finally, differences between the results in the intervention and the control group are relatively small. However, the results in regard to changes of cardiometabolic risk factors are consistent with changes of the body composition.

Among older people at high and very high cardiovascular risk, the conclusion is that the addition of telehealth strategies using an initial comprehensive counselling session followed by remote support via phone calls from a trained nurse over a period of three months resulted in improvements of cardiovascular risk factors and body composition. These improvements have been sustained after one year. These results indicate that telehealth strategies may offer an advantage over standard institution-based interventions.

## Data Accessibility Statements

Data associated with this paper is available by request from the corresponding author.
